# Analysis of *Tamarix chinensis* Forest Characteristics, Salt Ion Distribution, and Non-Structural Carbohydrate Levels in the Yellow River Delta: A Spatial Study Based on Proximity to the Shoreline

**DOI:** 10.3390/plants13172372

**Published:** 2024-08-26

**Authors:** Peili Mao, Qingzhi Lin, Banghua Cao, Jiabao Qiao, Kexin Wang, Xin Han, Yuanxiang Pang, Xiaonan Cao, Bo Jia, Qingshan Yang

**Affiliations:** 1 State Forestry and Grassland Administration Key Laboratory of Silviculture in Downstream Areas of the Yellow River, Shandong Agricultural University, Tai’an 271018, China; maopl1979@163.com (P.M.); lin15634195020@163.com (Q.L.); 17866710693@163.com (J.Q.); wkxsdau@163.com (K.W.); pangyuanxiang2020@126.com (Y.P.); caoxiaonan_05@163.com (X.C.); jiabo@sdau.edu.cn (B.J.); 2 Shandong Academy of Forestry, Jinan 250014, China; 15315050868@163.com

**Keywords:** growth potential, soil salt content, soil moisture content, ion balance, non-structural carbohydrates

## Abstract

The distribution of vegetation in coastal wetlands is significantly influenced by soil properties. However, the mechanisms of how soil characteristics impact the physiological processes of *Tamarix chinensis* forests remain underexplored. This study examined changes in the soil physicochemical properties and structural attributes of natural *T. chinensis* forests in the Yellow River Delta with increasing distance from the shoreline. *T. chinensis* trees were classified into healthy, intermediate, and dying categories based on growth potential, and dynamic changes in salt ions and non-structural carbohydrates (NSCs) were investigated. Results indicated that increasing distance from the shoreline corresponded to decreased soil salinity and pH, and increased soil moisture. *T. chinensis* mortality rate decreased, while tree height and ground diameter increased with distance. Soil salt content was positively correlated with *T. chinensis* mortality, but negatively correlated with tree height and ground diameter. Trees with lower growth potential had higher Na^+^ but lower K^+^ and K^+^/Na^+^ ratio. Soil salt content was positively correlated with root and stem Na^+^, while soil moisture was positively correlated with leaf NSCs. These findings suggest that soil salt content and moisture significantly influence *T. chinensis* ion absorption and NSC accumulation, with sodium toxicity being a key factor in the spatial distribution of *T. chinensis* forests.

## 1. Introduction

Coastal wetlands, situated at the interface of terrestrial and marine ecosystems, are characterized by an environmental gradient strongly influenced by tidal and salinity dynamics, which vary markedly with elevation. Areas with frequent tidal effects often have higher salinity; in areas with less tidal action, salinity is often lower [1]. With increasing distance from the ocean, soil nutrients gradually decrease [2]. Soil salinity has a significant impact on soil characteristics [3,4,5]. Soil salinization alters soil aggregate structure, hinders the soil nutrient transformation process, diminishes the effective nutrient release rate, and reduces the organic matter content [6]. Moreover, the heightened soil salinity effects of increased salinity on soil nutrients may also occur through decrease in soil biodiversity and microbial activity [5]. The increase in soil salt content leads to an increase in soil pH, while excessively high soil pH leads to a decrease in enzyme activities, such as urease and phosphatase, and a decrease in microbial diversity [7]. The increase in electrical conductivity after soil salinization has a negative impact on soil structural stability, permeability, and bulk density. The increase in soil bulk density leads to difficulties in water transport and exacerbates the accumulation of salt [8]. Soil salinity is a crucial determinant of vegetation distribution in coastal wetlands [9,10]. Therefore, revealing the changes in soil salinity and other soil characteristics with increasing distance from the sea is of great significance for elucidating the distribution of vegetation in these ecologically significant areas.

Under salt stress, plant growth is significantly inhibited [11], while their height, leaf area, and organic matter accumulation decrease [12,13,14]. Non-halophytes are sensitive to salt stress and have poor salt tolerance [15,16]. Conversely, halophytes may experience growth enhancement in low-salinity conditions, but they too suffer growth inhibition under severe salt stress [17]. The ion toxicity in plant cells under salt stress involves a substantial increase in Na^+^ [18]. When the Na^+^ exceeds a certain threshold, ion toxicity occurs, and plants cannot maintain ion homeostasis and growth [19]. The potassium to sodium ratio (K^+^/Na^+^) is a critical parameter for photosynthesis and an important indicator of a plant’s salt tolerance [20,21]. Meanwhile, excessive Na^+^ inhibits the absorption and transport of Ca^2+^ and K^+^ plasma nutrients by plants, leading to a decrease in the K^+^/Na^+^ ratio and breaking of the ion balance [22,23,24]. The ability of plants to survive under salinity depends on their ability to maintain Na^+^ homeostasis [21,25,26]. And the reasonable distribution of Na^+^ in various tissues is one of the most important strategies for plants to resist salt stress [20,24,27]. Researchers have found that some plants retain most of the absorbed Na^+^ in their stems [28], some in their leaves [14], and some in their roots [16,26]. Therefore, there are different mechanisms by which plants regulate the storage of salt ions in different organs under salt stress.

Non-structural carbohydrates (NSCs), including soluble sugars and starch synthesized via photosynthesis and stored in the thin-walled cells of plant woody tissue, are vital for plant metabolic processes [29]. NSCs play an important regulatory role in maintaining the normal growth of plants in response to stress [30,31,32]. During adverse environmental conditions, starch can be converted into soluble sugars through hydrolysis, which increases the content of soluble sugars [30]. The increase in soluble sugars content improves the osmotic adjustment ability of plants [32]. Zhang et al. [33] noted that while short-term drought stress did not significantly alter NSC levels, prolonged and intense drought stress markedly reduced the NSC contents in young *Robinia pseudocacia* trees. Under suitable soil moisture content, the NSC content in plants gradually increases [34,35]. In saline environments, NSCs are considered a backup source of osmotic pressure or energy storage in saline environments [30,32]. The transportation of photosynthetic products under salt stress is significantly inhibited [21,36], but the NSC contents in plants decreases [14] and increases in other plants [21,37]. The diverse NSC responses in plants under salt stress may be an adaptive response to salt stress, or an “injury” response caused by insufficient carbon utilization due to growth arrest. Monitoring these changes is critical for a deeper understanding of the plant’s response to salt stress [30].

The Yellow River Delta, situated in northeastern Shandong Province, China, is a fan-shaped region formed through sedimentation, expansion, oscillation, and diversion of the Yellow River near its estuary. *Tamarix chinensis*, a perennial shrub or small tree, is the predominant species in this region, thriving primarily in elevated or distal seaside wetlands and inland saline-alkali soils. Research has found that water and salinity are key factors affecting the growth of *T. chinensis* forests in this area, leading to low quality of the forests [38]. In the transition from inland to coastal mudflat, the density of *T. chinensis* stands is uneven and the aggregation intensity increases. The crown of a tree also has varying degrees of withering, and the closer it is to the ocean, the greater the degree of withering, and the mortality rate of forest stands increases, characterized by increasingly simple composition of community species. At present, research on *T. chinensis* mainly focuses on forest soil nutrient characteristics [39], population distribution patterns [40], and stress resistance physiological characteristics [41,42,43]. However, research about the distribution mechanisms of natural *T. chinensis* forests on the scale of distance from the shoreline in the Yellow River Delta is not in-depth enough. This study selected a typical distribution area of *T. chinensis* from the Bohai Sea to the Yellow River, and investigated the forest characteristics, soil indicators, and individual physiological indicators of *T. chinensis* forests with increasing distance from the shoreline. The main objectives were as follows: (1) Revealing changes in forest structure, survival status, and soil characteristics in *T. chinensis* forests, and identifying the main soil factors that affect forest characteristics; (2) Analyzing the relationships between soil factors and the distribution of ions, such as Na^+^ and K^+^, and of NSCs in *T. chinensis*, and exploring the relationship between ion distribution and NSCs.

## 2. Results

### 2.1. Soil and Stand Characteristics of T. chinensis Forests with Increasing Distance to the Shoreline

#### 2.1.1. Soil Characteristics

**Figure 1:**
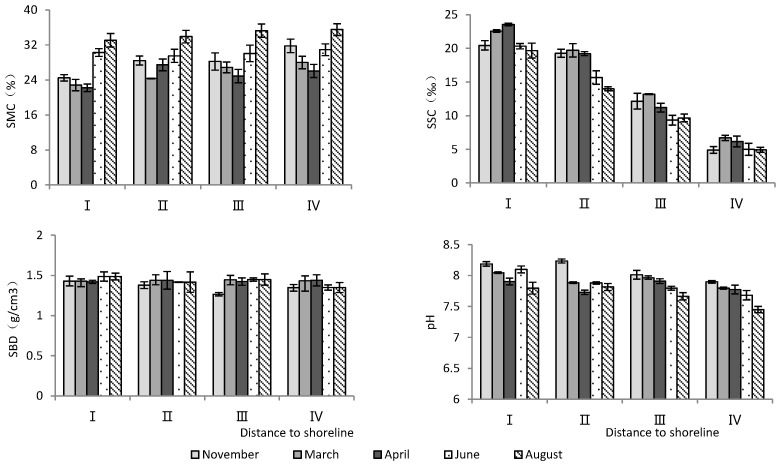
Monthly dynamic changes in soil indicators with increasing distance to shoreline. SMC, soil moisture content; SSC, soil salt content; SBD, soil bulk density.

According to Table 1, the distance to the shoreline and month had highly significant effects on soil water content, salinity, and pH, but not on soil bulk density (Table 1). As the distance to the shoreline increased, soil water content increased significantly, while soil salinity and pH decreased significantly (Figure 1). Temporally, June and August had the highest soil water content and the lowest soil salinity, while soil pH was the lowest in August (Figure 1).

#### 2.1.2. Stand Characteristics

- Growth characteristics

**Figure 2:**
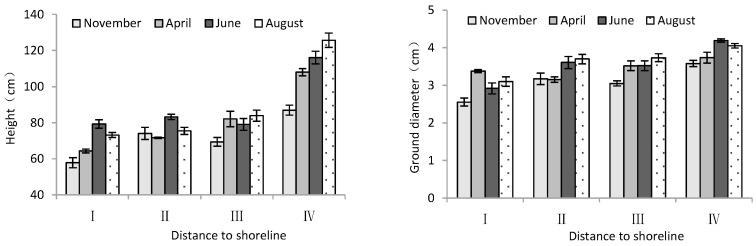
Height and ground diameter of *T. chinensis* with increasing distance to the shoreline.

According to Table 2, month significantly influenced the height and ground diameter of trees. The distance from the shoreline showed a highly significant effect on tree height and ground diameter of *T. chinensis*. Both tree height and ground diameter increased gradually with increase in distance from the shoreline. For tree height and diameter, site IV was significantly larger than site I (*p* < 0.05), and the difference between sample sites II and III was not significant (*p* > 0.05). Over the course of the months, both tree height and ground diameter showed an increasing trend with month (Figure 2), in which the tree height and ground diameter in June and August were significantly higher than those in November 2020 (*p* < 0.05).

- Mortality rate and growth potential

**Figure 3:**
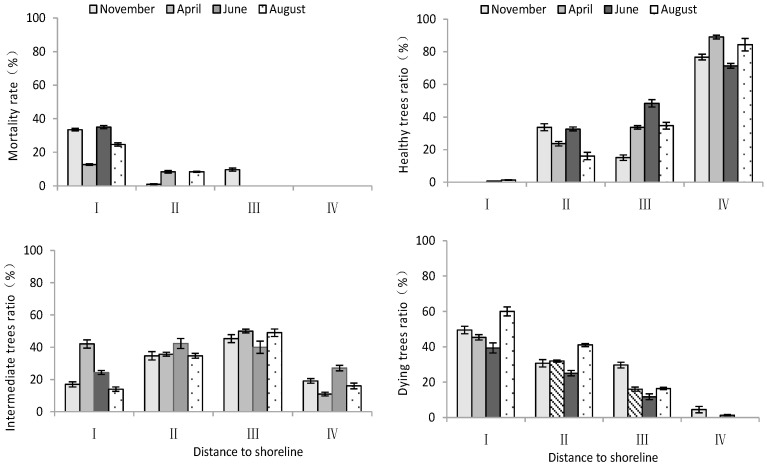
Changes in mortality rate and growth potential of *T. chinensis* with increasing distance to shoreline.

According to Table 2, distance to the shoreline had a highly significant effect on the mortality rate and growth potential of *T. chinensis*. However, the effects of month on the mortality rate and growth potential of *T. chinensis* were not significant. The mortality ratio and dying trees ratio of *T. chinensis* decreased with increase in distance to the shoreline (Figure 3), and site I was significantly higher than site IV (*p* < 0.05). Conversely, the healthy trees ratio of *T. chinensis* increased gradually with increase in distance from the shoreline. The healthy trees ratio in site IV was the largest and significantly higher than that of site I (*p* < 0.05), and the difference between sites II and III was not significant (*p* > 0.05).

### 2.2. Differences in Physiological Adaptations of T. chinensis of Different Growth Potentials with Increasing Distance to the Shoreline

#### 2.2.1. K^+^ and Na^+^ Contents in Roots, Stems, and Leaves

- K^+^ and Na^+^ content in root system

**Figure 4:**
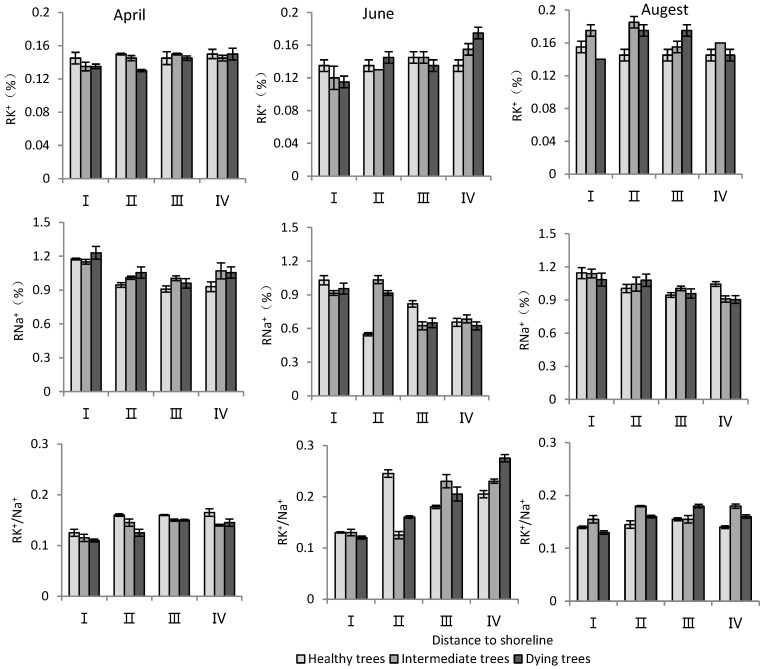
Monthly dynamic changes in K^+^ and Na^+^ contents in roots of *T. chinensis* with different growth potentials with increasing distance to shoreline. Root potassium content, RK^+^; Root sodium content, RNa^+^; Root potassium sodium ratio, RK^+^/Na^+^.

According to Table 3, distance to the shoreline and month had highly significant effects on the K^+^ and Na^+^ content and K^+^/Na^+^ ratio in the root system. The growth potential had a significant effect on the ion content and no significant effect on the K^+^/Na^+^ ratio. For the K^+^ content in the root system, it increased gradually with increasing distance from the shoreline (Figure 4). The K^+^ content of intermediate and dying trees was higher than that of healthy wood, and it was significantly higher in August than in April and June (*p* < 0.05). The root Na^+^ content increased with decrease in distance from the shoreline and growth potential, and the content in June was significantly lower than that in April and August (*p* < 0.05). Regarding the K^+^/Na^+^ ratio in the root system, a larger ratio was observed with increasing distance from the shoreline, and it was significantly higher in June compared to other months (Figure 4).

- K^+^ and Na^+^ content in stems

**Figure 5:**
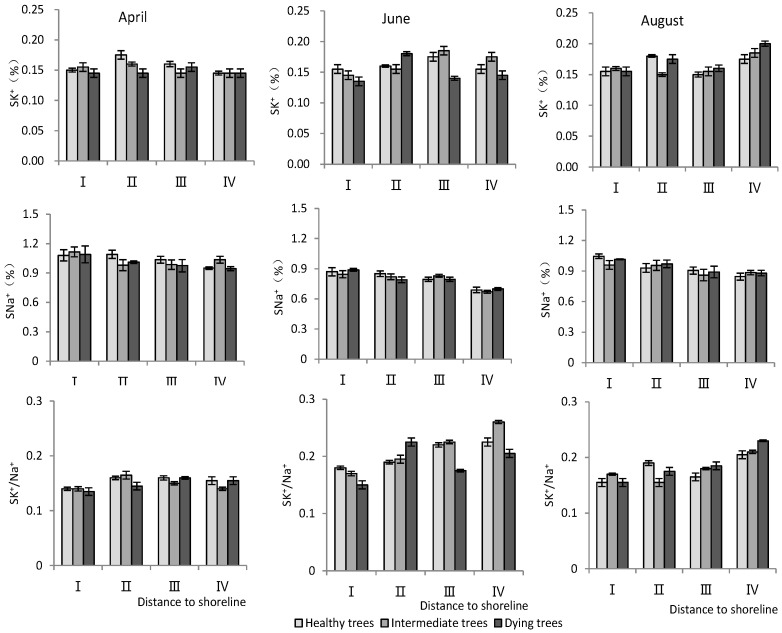
Monthly dynamic changes in K^+^ and Na^+^ contents in stem of *T. chinensis* with different growth potentials with increasing distance to shoreline. Stem potassium content, SK^+^; Stem sodium content, SNa^+^; Stem potassium sodium ratio, SK^+^/Na^+^.

According to Table 3, the distance to the shoreline and month had a highly significant effect on the ion content; the growth potential had a significant effect on K^+^, and had a non-significant effect on the Na^+^ content but a highly significant effect on the K^+^/Na^+^ ratio. For the K^+^ content, it was significantly higher in site IV than in site I (*p* < 0.05), and the K^+^ content decreased significantly (*p* < 0.05) with decrease in growth potential. And its content in August was significantly higher (*p* < 0.05) than that in June and April. For the Na^+^ content, it decreased significantly (*p* < 0.05) with increasing distance to the shoreline, with the maximum value in April. The K^+^/Na^+^ ratio increased with distance from the shoreline. Healthy wood showed a significantly higher K^+^/Na^+^ ratio than dying wood (*p* < 0.05). Among the months, June had the highest K^+^/Na^+^ ratio (Figure 5).

- K^+^ and Na^+^ content in leaves

**Figure 6:**
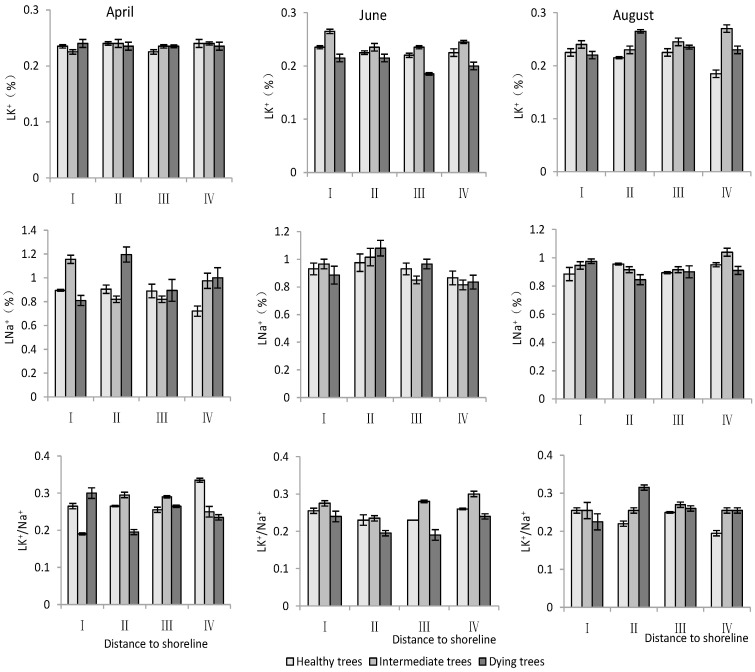
Monthly dynamic changes in K^+^ and Na^+^ contents in leaf of *T. chinensis* with different growth potentials with increasing distance to shoreline. Leaf potassium content, LK^+^; Leaf sodium content, LNa^+^; Leaf potassium sodium ratio, LK^+^/Na^+^.

According to Table 3, both the distance to the shoreline and growth potential had highly significant effects on K^+^ and Na^+^ contents as well as the K^+^/Na^+^ ratio in leaves, and month had highly significant effects on K^+^ contents and K^+^/Na^+^, but non-significant effects on Na^+^. For K^+^, the minimum value was in June and its content was significantly lower (*p* < 0.05) than that in April and August. Both K^+^ and Na^+^ contents decreased with increasing distance to the shoreline. The Na^+^ content was significantly lower (*p* < 0.05) in healthy trees than in intermediate and dying trees. The K^+^/Na^+^ ratio was significantly higher (*p* < 0.05) in site IV than in site I. And it was the smallest in dying wood, and significantly higher (*p* < 0.05) in April than in other months (Figure 6).

#### 2.2.2. NSC Content in Roots, Stems, and Leaves

- Soluble sugar and starch content in roots

**Figure 7:**
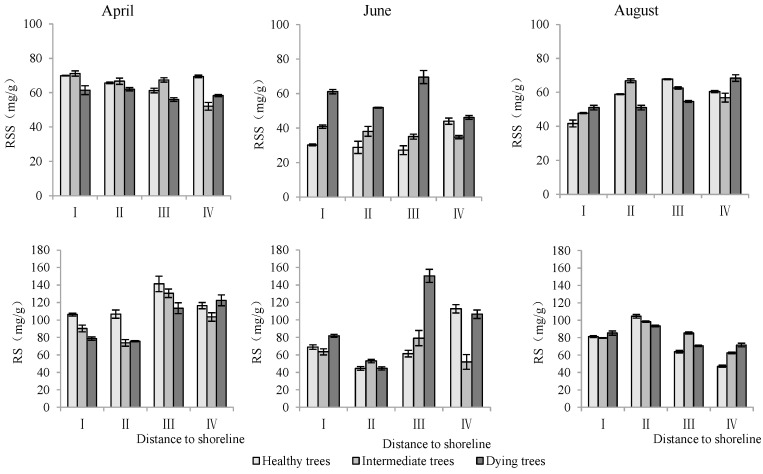
Monthly dynamics of root NSC content in *T. chinensis* with different growth potentials with increasing distance to shoreline. Root soluble sugar, RSS; Root starch, RS.

Distance to the shoreline, growth potential, and month all had highly significant effects on the soluble sugar and starch of the root system (*p* < 0.01) (Table 3). The soluble sugar and starch contents of the roots in sites III and IV, which were farther away from the shoreline, were significantly higher than those in I and II. Dying trees had significantly greater soluble sugar and starch contents than healthy and intermediate trees. The contents of soluble sugar and starch were significantly higher in April than those in June and August (*p* < 0.05) (Figure 7).

- Soluble sugar and starch content in stems

**Figure 8:**
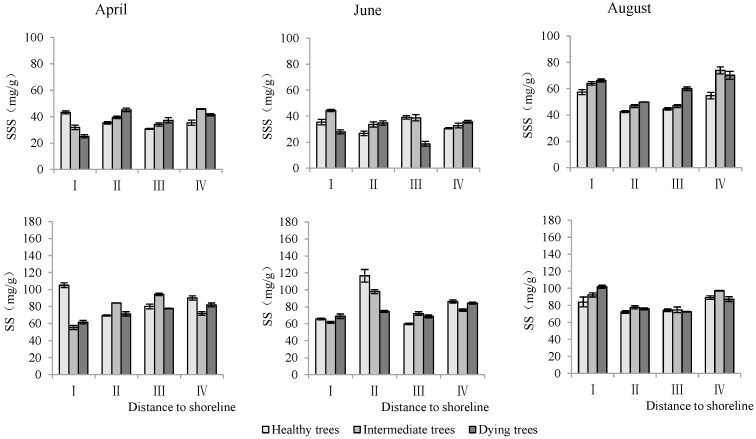
Monthly dynamics of stem NSC content in *T. chinensis* of different growth potentials with increasing distance to shoreline. Stem soluble sugar, SSS; Stem starch, SS.

As shown in Table 3, the distance to the shoreline, growth potential, and month each had highly significant effects on stem soluble sugar and starch (*p* < 0.01). The contents of soluble sugar and starch in stems were highest in site IV. The soluble sugar content in dying wood was significantly lower than that in intermediate and healthy trees, but the starch content showed the opposite trend. The soluble sugar content in August was significantly higher than that measured in April and June, but starch content was significantly lower than that in April and June (*p* < 0.05) (Figure 8).

- Soluble sugar and starch content in leaves

**Figure 9:**
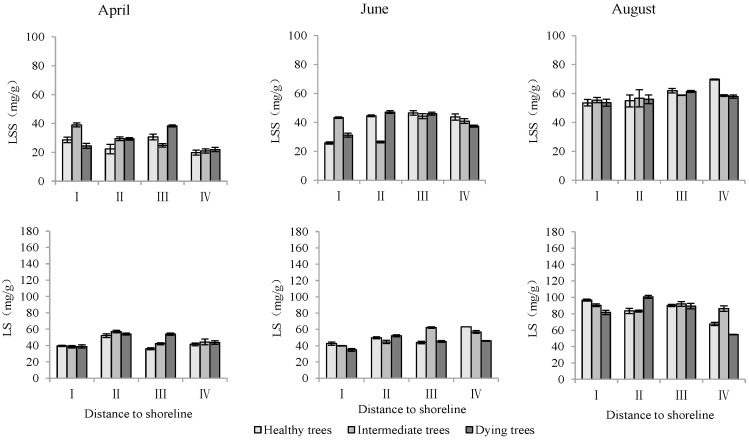
Monthly dynamics of leaf NSC content in *T. chinensis* of different growth potentials with increasing distance to shoreline. Leaf soluble sugar, LSS; Leaf starch, LS.

The distance from the shoreline and the growth potential did not significantly affect the leaf soluble sugar content (*p* > 0.05). However, the month had a significant impact on leaf soluble sugar levels (*p* < 0.05). Furthermore, the distance to the shoreline, growth potential, and month all had a highly significant influence on leaf starch content (*p* < 0.01). (Table 3). The starch contents in leaves in sites III and IV were significantly higher than those in sites I and II. The order of starch content in leaves with different growth potentials was as follows: dying trees > intermediate trees > healthy trees. Soluble sugar and starch contents measured in August were significantly greater than those measured in June and April (*p* < 0.05) (Figure 9).

### 2.3. The Correlation between Physiological Indicators, Stand Indicators, and Soil Indicators of T. chinensis

SSC exhibited a significant positive correlation with SBD and pH but showed no significant correlation with SMC. Meanwhile, SSC was significantly negatively correlated with H and D, significantly positively correlated with MR, SNa^+^, and RNa^+^, and significantly negatively correlated with the SK^+^/Na^+^ ratio and the RK^+^/Na^+^ ratio, but not significantly correlated with LNa^+^. RNa^+^ displayed a positive correlation with SNa^+^ and had a significant negative correlation with the SK^+^/Na^+^ ratio but was not significantly correlated with LNa^+^ (Table 4).

SMC was significantly positively correlated with LSS and LS, but was not significantly correlated with SSS, SS, LSS, and LS. LSS was positively correlated with LS, and both were positively correlated with SSS, but not significantly correlated with RSS and RS. And both SSS and SS were not significantly correlated with RSS and RS (Table 4).

LNa^+^ had a significant negative correlation with RS. The LK^+^/Na^+^ ratio was positively correlated with RS. SK^+^ was significantly negatively correlated with LSS and positively correlated with RSS. SNa^+^ was significantly positively correlated with RSS. RK^+^ was significantly positively correlated with LS. RNa^+^ was significantly positively correlated with RSS, while the RK^+^/Na^+^ ratio was negatively correlated with RSS (Table 4).

## 3. Discussion

The water tables get shallower as the distance from the shoreline decreases in coastal wetland and severe evaporation accumulates more salt ions on the surface, leading to an increase in salt content [44]. Along the Yellow River region, river runoff ameliorates soil salinity through drainage, underground seepage, and other means [45]. Our study indicated that the soil salinity at site I, nearest to the sea, was the highest, while site IV, closest to the Yellow River, exhibited the lowest soil salinity. Generally, the soil salinity in the region decreased from coastal to inland and increased from the Yellow River channel to both sides. Correlation analysis showed a positive correlation between soil salt content, soil bulk density, and pH, indicating that soil salt content was an important factor affecting soil characteristics [3,5,7]. Our study found that soil salinity was significantly negatively correlated with the tree height and ground diameter, and highly significantly positively correlated with the mortality rate of *T. chinensis*. The mortality rate of *T. chinensis* and the proportion of dying trees increased as the distance from the shoreline decreased, which was closely related to poor soil conditions resulting from high soil salinity [3,5]. In addition, we found *T. chinensis* had a strong salt tolerance, but when the soil salt content was high, the salt content increased significantly, and its cell membrane was severely damaged, and even the ground part died [34]. This supported that hypersalinity was an important cause of plant death in salt marshes [46,47]. Therefore, soil salinity was considered as a key environmental factor affecting the distribution of *T. chinensis* based on distance from the shoreline. Meanwhile, it was also found that the correlation between the soil moisture content and the leaf starch content was positive, which was related to the enhanced photosynthesis of trees under appropriate soil moisture [48]. Higher soil moisture was associated with increased soil nitrogen content [2], a crucial factor promoting plant growth. Furthermore, the increase in starch content enhanced the ability of plants to adapt to adversity [49]. Therefore, soil salinity and moisture affect the distribution of *T. chinensis*.

The regulation of Na^+^ under salt stress was a reflection of plant salt tolerance, as indicated in studies by Plett and Møller [25] and Acosta-Motos et al. [26]. The closer to the shoreline, the higher the salt content in the soil would be. K^+^ content in the *T. chinensis* was relatively stable and Na^+^ gradually increased closer to the shoreline. Between different months, the Na^+^ content in *T. chinensis* was the highest in April. Correlation analysis showed that the Na^+^ content in the roots and stems of *T. chinensis* was positively correlated with soil salt content. Therefore, the changes in Na^+^ content in *T. chinensis*, in terms of distance from the sea and month, were significantly influenced by soil salinity. The ion toxicity in plant cells under salt stress was caused by a significant increase in Na^+^, which had adverse effects on cytoplasmic enzyme activity, photosynthesis, and metabolism [18]. The K^+^/Na^+^ ratio is an important indicator for reflecting the salt tolerance of plants, and maintaining a high value is likely one of the key determinants of plant salt tolerance [11,23,27]. In this study, a lower K^+^/Na^+^ ratio was observed in *T. chinensis* closer to the shoreline and in poorer growth conditions. Therefore, the imbalance of K^+^/Na^+^ caused significant ion toxicity to *T. chinensis*, which was an important reason for the short stature and more decaying trees of *T. chinensis* plants. Growth potential showed no significant effects on RK^+^/Na^+^, but SK^+^/Na^+^ and LK^+^/Na^+^ of dying wood were lower than that of healthy wood. The root system is the main organ that regulates the entry of ions into the body [26,50], and our results also showed that Na^+^ content in the root system was positively correlated with the soil salt content. Some halophytes, including *T. chinensis*, can accumulate Na^+^ in leaves through transpiration and subsequently excrete it using salt glands [51]. It was also found that the Na^+^ content in the root system was positively correlated with that in the stem, but the correlation with the Na^+^ content in the leaves was not significant. The correlation analysis showed that LK^+^/Na^+^ was significantly negatively correlated with LNa^+^. This might be related to the use of salt gland tissue to excrete Na^+^ from the plant, increasing LK^+^/Na^+^. Therefore, the regulation of sodium and potassium ions by different organs may be an important mechanism for *T. chinensis* to adapt to coastal salt marshes.

Maintaining high NSC levels within plants under adverse conditions enhanced their survival ability [32,49,52]. The NSC contents of trees showed significant changes with phenological changes [53]. It was found that the NSC content in the above-ground leaves and stems of *T. chinensis* was the lowest in April and the highest in August during the early stage of leaf expansion, which was related to the increased photosynthetic capacity of plants with environmental improvement [53,54]. The NSC contents in the roots of *T. chinensis* initially decreased and then increased from April to August, showing no direct correlation with leaf NSC content. Newell et al. [54] also found no correlation between NSC in roots and in the leaves of trees. This phenomenon might result from the delayed transport response of photosynthetic products in leaves, as the root was the main storage organ of NSC. As the distance to the shoreline increased, the NSC content in *T. chinensis* gradually increased, which was related to the increase in photosynthetic capacity caused by the decrease in soil salt content [43,55]. This study demonstrated that the NSC contents gradually increased with decline in growth potential. This was probably due to the inhibition of sucrose transport by Na^+^ [21]. Dying trees have the highest soluble sugar content in the roots, stems, and leaves, which improves their osmotic regulation ability [32] and repair of embolisms [52]. Soluble sugars and starches in leaves and stems had no correlation with those in roots, which might be related to the transport inhibition of photosynthetic products. Limited photosynthetic products were preferentially stored under stress, which may result from survival relying more on carbon metabolism than growth [56]. Root soluble sugars were significantly positively correlated with root Na^+^ and negatively correlated with the root K^+^/Na^+^ ratio, indicating that soluble sugars participated in the absorption of K^+^ and Na^+^ [14,57]. However, further study is needed to investigate how NSC affects ion absorption.

## 4. Conclusions

This study demonstrated that the physical and chemical properties of soil and the growth of *T. chinensis* improved significantly with increase in distance from the shoreline, which was closely related to the decrease in soil salt content. The closer the distance from the shoreline, the worse the growth potential, the higher the Na^+^ content in *T. chinensis*, and the lower the K^+^/Na^+^ ratio, so the ion toxicity was an important reason for the decline in *T. chinensis* forest. The NSCs of *T. chinensis* increased with increase in distance from the shoreline, but the NSCs of dying trees were higher than those of healthy trees and were preferentially used for storage. Moreover, it was found that soluble sugar in the root system was involved in the absorption of K^+^ and Na^+^. As a consequence, the spatiotemporal variation of Na^+^ affects the level of ion toxicity and growth of *T. chinensis*, which is one of the key factors determining its natural distribution. With the change in global climate, changes in precipitation patterns and increased temperatures will significantly affect soil water–salt change in coastal wetlands, which will result in significant impacts on the distribution of vegetation. Therefore, it is necessary to strengthen the long-term positional monitoring of *T. chinensis* forests in the region to provide a theoretical foundation for eco-environment construction in coastal wetlands.

## 5. Materials and Methods

### 5.1. Study Area

The Yellow River Delta has a warm temperate monsoon continental climate with four distinct seasons: a cold winter, a hot summer with concurrent rainfall, a dry and windy spring, and a fall with sharply reduced rainfall. The climate features of spring drought, summer flood and late autumn drought are formed in this area. The average annual temperature is 12.3 °C, the average frost-free period is 210 days, and the average annual precipitation is 559 mm, 70% of which is distributed in summer. Due to the influence of the Yellow River, the impact of flooding deposition is mainly due to the coastal tidal saline soil, which has a high salt content, and grows various types of halophytic vegetation depending on the salt content. The main vegetation types in the Yellow River Delta region include scrub and saline meadows, with prevalent species such as *Suaeda salsa*, *Miscanthus sacchariflorus*, *Phragmites australis*, *T. chinensis*, etc.

### 5.2. Test Site

**Figure 10:**
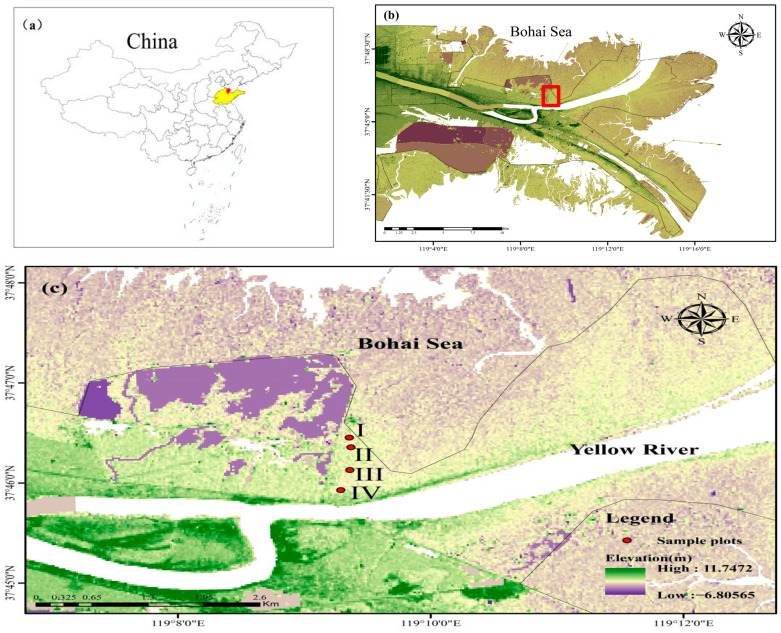
Geographical location of the study area in China (**a**) and Yellow River Delta National Nature Reserve (**b**) and experiment sites (**c**).

The experimental forest is located at the Yellow River Delta National Nature Reserve. Based on the distribution of natural *T. chinensis* forests in relation to the distance from the shoreline, along the direction perpendicular to the coastal zone, we established four experimental gradients representing different distances from the coastline, corresponding to four sampling sites (i.e., I, II, III, and IV in Figure 10 and Table 5). The side closest to the shoreline was sequentially numbered as sample sites I, II, III, and IV (Figure 10). On each sample site, three sampling plots of 5 m × 5 m were selected as replicates. Each plot was spaced by at least 30 m. According to laboratory measurements, the soil nutrients in this area were as follows: total nitrogen at 0.27 g/kg, hydrolyzable nitrogen at 7.33 mg/kg, available phosphorus at 5.89 mg/kg, available potassium at 172.2 mg/kg, and organic matter at 4.5 g/kg.

### 5.3. Experimental Design

A field survey was conducted in November 2020, and subsequently in April, June, and August of 2021. Three soil samples of 0–20 cm in each sampling plot were collected randomly to determine soil moisture content, soil salt content, soil bulk density, and soil pH, with a total of 36 samples. The number of *T. chinensis* plants, their height, diameter, and number of dead plants in the sample plots were measured. According to the proportion of the total number of dead branches in the tree crown, the growth trend was divided into healthy trees with a ratio of ≤1/3, intermediate trees with a ratio of ≤2/3, and dying trees with a ratio of >2/3. The ratio of trees with different growth potential was equal to the number of trees with different growth potential divided by the total number of all trees in the corresponding survey sample multiplied by 100%. The NSCs of plants are mainly composed of soluble sugars and starch [58]. In April, June, and August 2021, samples of roots, stems, and leaves of 5 individual plants per growth potential were collected to determine the contents of soluble sugars, starch, Na^+^, and K^+^.

### 5.4. Indicator Measurement

#### 5.4.1. Soil Indicators

Soil moisture content (SMC) was measured by drying method; soil salt content (SSC) was measured using the conductivity method, and the standard curve of soil salt concentration was measured using the drying residue method with a conductivity meter; soil pH was measured by a pH meter [59]. Additionally, soil bulk density (SBD) was measured using the core cutter or cutting ring method [60].

#### 5.4.2. Physiological Indicators

Soluble sugar and starch content were determined by anthrone colorimetry [61], and the contents of Na^+^ and K^+^ were determined by water bath extraction using a mixture of perchloric acid and concentrated nitric acid [62].

### 5.5. Data Analysis

Two-factor ANOVA was used to compare the effects of month (investigation time) and distance to the shoreline on the stand characteristics of *T. chinensis* forests. Three-factor ANOVA was used to compare the effects of month, distance to shoreline, and growth potential on physiological indicators. If the differences were significant, multiple comparisons were made using the least significant difference method (LSD), and the level of significant difference test in the analysis was *p* < 0.05. Correlation analyses were carried out between the soil characteristics of the field survey and the characteristics of the *T. chinensis* forests. All analyses were conducted using Excel 2010 and SPSS 23.0 software.

## Figures and Tables

**Table 1:** Results of two-way ANOVA of the impact of month (investigation time) and distance to the shoreline on soil characteristics. SMC, soil moisture content; SSC, soil salt content; SBD, soil bulk density.

Indexes	Investigation Time (A)		Distance to Shoreline (B)		A × B	
	F Value	*p* Value	F Value	*p* value	F Value	*p* Value
SMC	7.46	<0.01	9.68	<0.01	1.89	0.07
SSC	100.65	<0.01	30.24	<0.01	13.97	<0.01
SBD	1.64	0.18	1.40	0.26	0.71	0.73
pH	11.40	<0.01	9.41	<0.01	1.10	0.39

**Table 2:** Results of two-way ANOVA of the effects of month (investigation time) and distance to the shoreline on the stand characteristics of *T. chinensis*.

Indexes	Investigation Time (A)		Distance to Shoreline (B)		A × B	
	F Value	*p* Value	F Value	*p* Value	F Value	*p* Value
Height	7.40	<0.01	36.04	<0.01	1.47	0.20
Ground diameter	3.84	<0.05	8.93	<0.01	0.70	0.70
Mortality rate	2.63	0.05	74.47	<0.01	5.62	<0.01
Healthy trees ratio	0.47	0.70	58.78	<0.01	1.29	0.28
Intermediate trees ratio	0.52	0.68	8.29	<0.01	0.87	0.56
Dying trees ratio	2.55	0.06	55.51	<0.01	1.12	0.35

**Table 3:** Variance analysis results of sea distance, growth potential, and investigation time on physiological indexes of *T. chinensis*.; Root potassium content, RK^+^; Root sodium content, RNa^+^; Root potassium sodium ratio, RK^+^/Na^+^; Stem potassium content, SK^+^; Stem sodium content, SNa^+^; Stem potassium sodium ratio, SK^+^/Na^+^; Leaf potassium content, LK^+^; Leaf sodium content, LNa^+^; Leaf potassium sodium ratio, LK^+^/Na^+^; Root soluble sugar, RSS; Root starch, RS; Stem soluble sugar, SSS; Stem starch, SS; Leaf soluble sugar, LSS; Leaf starch, LS.

Indexes	Investigation Time (A)		Distance from Sea (B)		Growth Potential (C)		A × B		A × C		B × C		A × B × C	
	F Value	*p* Value	F Value	*p* Value	F Value	*p* Value	F Value	*p* Value	F Value	*p* Value	F Value	*p* Value	F Value	*p* Value
RK^+^	55.84	<0.01	11.30	<0.01	4.74	<0.05	11.75	<0.01	10.21	<0.01	5.56	<0.01	7.03	<0.01
RNa^+^	238.14	<0.01	93.69	<0.01	4.31	<0.05	6.79	<0.01	3.62	<0.05	11.71	<0.01	0.30	0.56
RK^+^/Na^+^	129.34	<0.01	102.44	<0.01	0.38	0.69	26.58	<0.01	11.07	<0.01	12.83	<0.01	0.76	0.66
SK^+^	38.38	<0.01	21.61	<0.01	3.88	<0.05	20.65	<0.01	11.69	<0.01	7.99	<0.01	9.72	<0.01
SNa^+^	185.24	<0.01	39.49	<0.01	0.64	0.53	2.36	0.05	0.79	0.54	1.11	0.38	1.51	0.17
SK^+^/Na^+^	442.42	<0.01	157.23	<0.01	5.35	<0.01	30.89	<0.01	16.31	<0.01	5.81	<0.01	18.62	<0.01
LK^+^	15.28	<0.01	4.13	<0.05	51.47	<0.01	7.16	<0.01	40.31	<0.01	9.34	<0.01	8.44	<0.01
LNa^+^	0.05	0.95	9.05	<0.01	5.47	<0.01	7.47	<0.01	5.82	<0.01	9.64	<0.01	9.35	<0.01
LK^+^/Na^+^	15.32	<0.01	4.70	<0.01	18.97	<0.01	16.42	<0.01	35.98	<0.01	9.75	<0.01	25.47	<0.01
RSS	50.71	<0.01	59.82	<0.01	60.44	<0.01	88.47	<0.01	23.46	<0.01	38.97	<0.01	43.37	<0.01
RS	21.57	<0.01	66.13	<0.01	7.65	<0.01	79.98	<0.01	41.29	<0.01	20.98	<0.01	20.81	<0.01
SSS	1560.38	<0.01	107.31	<0.01	56.77	<0.01	83.38	<0.01	60.62	<0.01	35.25	<0.01	34.17	<0.01
SS	847.16	<0.01	577.65	<0.01	31.24	<0.01	852.63	<0.01	33.12	<0.01	38.37	<0.01	55.93	<0.01
LSS	5.03	<0.05	0.94	0.43	1.04	0.37	0.97	0.46	0.92	0.46	1.19	0.33	0.97	0.49
LS	1358.82	<0.01	125.37	<0.01	35.13	<0.01	370.30	<0.01	57.11	<0.01	182.44	<0.01	138.72	<0.01

**Table 4:** Correlation analysis between stand characteristics, physiological adaptability, and soil characteristics of *T. chinensis*. Tree height, H; Ground diameter, D; Mortality rate, MR; Leaf potassium content, LK^+^; Leaf sodium content, LNa^+^; Leaf potassium sodium ratio, LK^+^/Na^+^; Stem potassium content, SK^+^; Stem sodium content, SNa^+^; Stem potassium sodium ratio, SK^+^/Na^+^; Root potassium content, RK^+^; Root sodium content, RNa^+^; Root potassium sodium ratio, RK^+^/Na^+^; Leaf soluble sugar, LSS; Leaf starch, LS; Stem soluble sugar, SSS; Stem starch, SS; Root soluble sugar, RSS; Root starch, RS; Soil moisture content, SMC; Soil salinity content, SSC; Soil bulk density, SBD. *, *p* < 0.05; **, *p* < 0.01.

	H	D	MR	LK^+^	LNa^+^	LK^+^/Na^+^	SK^+^	SNa^+^	SK^+^/Na^+^	RK^+^	RNa^+^	RK^+^/Na^+^	LSS	LS	SSS	SS	RSS	RS	SMC	SSC	SBD
H	1.00																				
D	0.77 **	1.00																			
MR	−0.46	−0.79 **	1.00																		
LK^+^	−0.15	−0.28	0.31	1.00																	
LNa^+^	−0.25	−0.34	0.16	0.15	1.00																
LK^+^/Na^+^	0.17	0.10	0.10	0.39	−0.81 **	1.00															
SK^+^	−0.19	−0.18	−0.16	0.26	−0.02	0.30	1.00														
SNa^+^	−0.56	−0.53	0.28	0.49	0.22	0.17	0.71 *	1.00													
SK^+^/Na^+^	0.63 *	0.63 *	−0.41	−0.60 *	−0.06	−0.35	−0.66 *	−0.91 **	1.00												
RK^+^	0.22	0.56	−0.41	0.02	−0.42	0.32	−0.18	−0.03	0.11	1.00											
RNa^+^	−0.44	−0.48	0.46	0.65 *	0.26	0.21	0.44	0.90 **	−0.84 **	0.05	1.00										
RK^+^/Na^+^	0.48	0.65 *	−0.55	−0.69 *	−0.37	−0.13	−0.47	−0.86 **	0.84 **	0.29	−0.92 **	1.00									
LSS	0.17	0.33	−0.08	−0.12	0.05	−0.25	−0.74 **	−0.30	0.47	0.53	−0.07	0.21	1.00								
LS	−0.01	0.17	−0.01	0.20	−0.05	0.02	−0.51	−0.01	0.11	0.75 **	0.19	0.03	0.84 **	1.00							
SSS	0.29	0.11	0.12	0.26	0.17	−0.02	−0.29	0.17	0.06	0.45	0.39	−0.26	0.69 *	0.74 **	1.00						
SS	0.37	0.32	−0.27	−0.05	0.31	−0.25	−0.08	−0.05	0.21	0.35	0.03	0.09	0.20	0.21	0.40	1.00					
RSS	−0.10	0.03	−0.20	0.49	−0.02	0.33	0.68 *	0.73 **	−0.54	0.19	0.65 *	−0.58 *	−0.08	0.09	0.26	−0.14	1.00				
RS	−0.10	0.01	−0.17	−0.06	−0.69 *	0.64 *	0.57	0.36	−0.43	0.25	0.11	−0.04	−0.43	−0.21	−0.29	−0.29	0.35	1.00			
SMC	0.33	0.29	0.01	0.05	0.02	−0.15	−0.83 **	−0.48	0.55	0.44	−0.22	0.29	0.88 **	0.80 **	0.68 *	0.16	−0.26	−0.51	1.00		
SSC	−0.84 **	−0.84 **	0.72 **	0.30	0.47	−0.19	0.20	0.59 *	−0.63 *	−0.42	0.60 *	−0.66 *	−0.25	−0.12	−0.15	−0.19	0.06	−0.17	−0.36	1.00	
SBD	−0.70 *	−0.85 **	0.64 *	0.29	0.15	−0.02	−0.01	0.39	−0.60 *	−0.33	0.39	−0.51	−0.16	0.05	−0.03	−0.36	−0.13	0.06	−0.09	0.59 *	1.00
pH	−0.65 *	−0.69 *	0.59 *	0.14	0.01	0.09	0.12	0.22	−0.51	−0.53	0.19	−0.34	−0.52	−0.47	−0.61 *	−0.37	−0.28	0.20	−0.51	0.64 *	0.64 *

**Table 5:** Overview of typical sample plots in the Yellow River Delta at different sea distances.

Sample Plot	Longitude and Latitude Coordinates	Distance to Shoreline (km)	Mixed Plant	Dominant Plant	Coverage
I	119.156674 E, 37.773742 N	3.1	None	*T. chinensis*	0.25
II	119.156501 E, 37.772322 N	3.5	None	*T. chinensis*	0.47
III	119.156163 E, 37.769495 N	4.0	*S. salsa*	*T. chinensis*	0.24
IV	119.154998 E, 37.763511 N	4.9	*S. salsa*, *Phragmites australis*, *Artemisia*	*T. chinensis*	0.51

## Data Availability

The original contributions presented in the study are included in the article. Further inquiries can be directed to the corresponding author.
